# Prognostic Factors and Survival in Surgically Treated Stage III Non-Small Cell Lung Cancer: A Real-World Single-Center Retrospective Cohort Study

**DOI:** 10.3390/curroncol33060316

**Published:** 2026-05-28

**Authors:** Bekir Elma, Hilal Zehra Kumbasar, Ilkay Dogan, Ahmet Ulusan, Maruf Sanli, Ahmet Ferudun Isik

**Affiliations:** 1 Department of Thoracic Surgery, Faculty of Medicine, Gaziantep University, 27310 Gaziantep, Türkiye; ulusan@gantep.edu.tr (A.U.); sanli@gantep.edu.tr (M.S.); aisik@gantep.edu.tr (A.F.I.); 2 Department of Thoracic Surgery, Alanya Training and Research Hospital, 07400 Antalya, Türkiye; hilal.kumbasar@saglik.gov.tr; 3 Department of Biostatistics, Faculty of Medicine, Gaziantep University, 27310 Gaziantep, Türkiye; ilkaydogan@gantep.edu.tr

**Keywords:** stage III NSCLC, upfront surgery, prognosis, survival analysis, nomogram, lymph node metastasis, thoracic oncology

## Abstract

**Background:** Stage III non-small cell lung cancer (NSCLC) represents a clinically and biologically heterogeneous disease group, posing challenges in treatment standardization. **Objective:** This study aimed to evaluate survival outcomes and prognostic factors in patients with pathological stage III NSCLC undergoing upfront surgery in a real-world setting. **Methods:** Patients treated between January 2009 and December 2023 were retrospectively analyzed. All patients underwent anatomical resection with systematic mediastinal lymph node dissection and achieved complete (R0) resection. Overall survival (OS) and recurrence-free survival (RFS) were estimated using the Kaplan–Meier method, and prognostic factors were evaluated using Cox proportional hazards regression. Nomograms were developed for individualized survival prediction. **Results:** A total of 390 patients were included, with a median follow-up of 35 months. The median OS was 47 months, and the median RFS was 30 months. Multivariable analysis identified age, PET-detected mediastinal nodal involvement, tumor location, postoperative complications, pathological nodal status, adjuvant chemotherapy, and adjuvant radiotherapy as independent predictors of OS (*p* = 0.001, *p* = 0.029, *p* = 0.006, *p* =0.002, *p* = 0.009, and 0.004, respectively), while adjuvant chemotherapy was associated with improved survival (*p* = 0.010). For RFS, pathological N2b disease, stage IIIB, tumor location, and adenocarcinoma histology were independent predictors (*p* = 0.001, *p* = 0.016, *p* = 0.029, and *p* = 0.027, respectively). The OS nomogram demonstrated moderate discriminative ability, whereas the RFS model showed limited predictive performance. **Conclusions:** These findings highlight the substantial heterogeneity of stage III NSCLC and emphasize the importance of individualized, multidisciplinary treatment strategies beyond conventional staging systems.

## Simple Summary

Stage III non-small cell lung cancer (NSCLC) includes a highly diverse group of patients with different clinical behaviors and outcomes. Although current treatment guidelines favor non-surgical approaches combined with systemic therapies, surgery is still performed in selected patients in real-world practice. This study evaluated patients who underwent upfront surgery to better understand survival differences and identify factors affecting prognosis. The results showed considerable variability in outcomes despite similar staging, highlighting limitations of traditional classification systems. These findings suggest that treatment decisions in stage III NSCLC should not rely solely on standardized algorithms but should instead incorporate individualized risk assessment and multidisciplinary evaluation.

## 1. Introduction

Stage III non-small cell lung cancer (NSCLC) is marked by significant clinical and biological heterogeneity. Treatment strategies at this stage require careful patient selection and multidisciplinary assessments. Guidelines prioritize consolidation immunotherapy after concurrent chemoradiotherapy for unresectable stage III, favoring non-surgical multimodal treatments [1,2,3]. Nevertheless, there is a continuing need to define which patients can benefit from surgical intervention.

The lack of clear definitions for “resectable” versus “unresectable” disease in stage III NSCLC leads to variability in surgical choices. Decisions depend on nodal involvement, patient status, and institutional experience, indicating that standard algorithms may not suit all stage III cases [4,5].

Thus, historical series have shown that upfront surgery can result in significant long-term survival for well-selected patients with stage III NSCLC [6,7,8]. Complete resection (R0) and limited mediastinal nodal disease are key factors.

Although nomograms and risk models have been developed to predict survival in stage III NSCLC, their accuracy at the individual level remains limited [9,10,11]. This underscores that clinical and biological heterogeneity cannot be fully addressed by clinical–pathological variables alone. This study evaluates survival outcomes and prognostic factors in stage III NSCLC patients undergoing upfront surgery, aiming to contextualize the role of surgery using real-world data.

However, despite advances in multimodal treatment strategies, the marked heterogeneity of stage III NSCLC continues to challenge treatment standardization, and conventional staging systems remain insufficient to fully capture individual prognostic variability.

## 2. Materials and Methods

### 2.1. Ethics Approval

The study was conducted in accordance with the Declaration of Helsinki and approved by the Clinical Research Ethics Committee of Gaziantep University (Approval No: 2025/431; Date: 7 January 2026). Due to the retrospective design and anonymized data collection, informed consent was waived.

### 2.2. Study Design and Patient Selection

This retrospective, single-center study included patients with stage III NSCLC who underwent upfront surgical resection between January 2009 and December 2023. All were staged using the 9th edition of the Tumor–Node–Metastasis (TNM) classification. The study period covers the pre-immunotherapy era; patients who received immunotherapy were excluded because it was infrequently and inconsistently used at the time.

**Figure 1:**
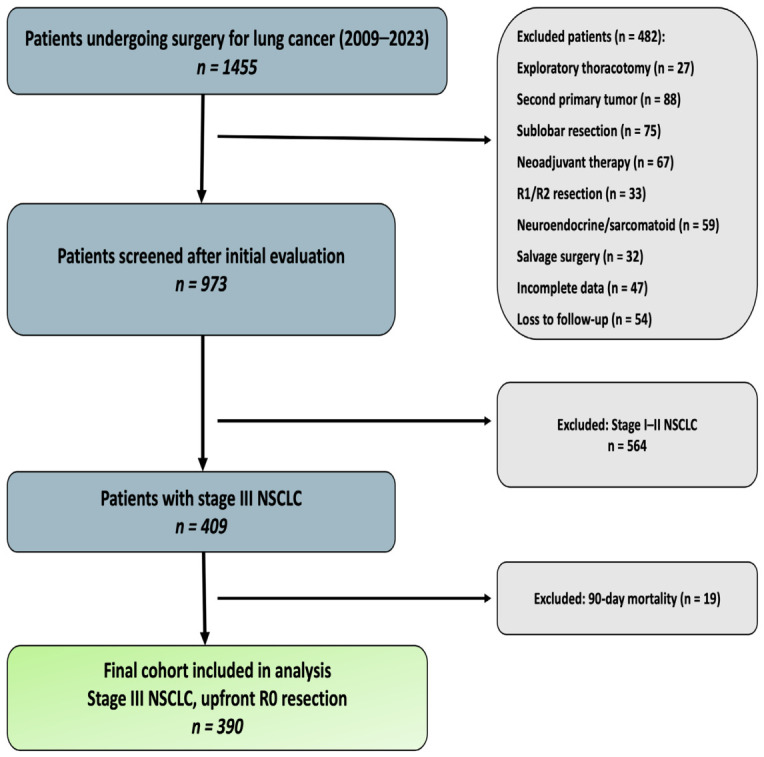
Flowchart of patient selection and study cohort formation.

Eligible patients had anatomical lung resection (lobectomy or pneumonectomy) and systematic mediastinal lymph node dissection per European Society of Thoracic Surgeons guidelines. Only those with R0 resection were included. We excluded patients with neuroendocrine or sarcomatoid carcinoma, exploratory thoracotomy without resection, neoadjuvant therapy before surgery, salvage surgery, a second primary malignancy, incomplete records, or missing follow-up data. Patients who died within 90 days after surgery were excluded to focus on long-term oncologic outcomes. After exclusions, the study included patients with pathological T1-4N0-2M0 who had upfront surgery. A flowchart illustrating patient selection is presented in Figure 1.

### 2.3. Preoperative Evaluation and Surgical Strategy

All patients had thoracic computed tomography and positron emission tomography (PET) for staging. Mediastinal lymph node positivity was defined as a short-axis diameter of at least 10 mm on computed tomography or a maximum standardized uptake value greater than 2.5 on PET.

Patients without mediastinal lymph node involvement or with only N1 disease were offered surgery directly. Those with suspected single-station N2 disease had endobronchial ultrasonography and/or mediastinoscopy for confirmation. If N2 was confirmed at a single station, upfront resection was considered. Patients with multi-station N2 disease were not operated on and were excluded.

Despite comprehensive preoperative staging, discrepancies between clinical and pathological nodal status were observed in a subset of patients, reflecting known limitations of current staging modalities.

### 2.4. Postoperative Management and Follow-Up

Postoperative pathology was discussed at the tumor board, and adjuvant treatment decisions (chemotherapy or chemoradiotherapy) were made based on results. Follow-up included thoracic computed tomography, PET, and brain magnetic resonance imaging if needed. Overall survival (OS) was defined as the time from the date of surgery to death from any cause or last follow-up. Recurrence-free survival (RFS) was defined as the time from surgery to the first documented local or distant recurrence. The last follow-up date for survival analyses was 15 January 2026.

### 2.5. Statistical Analysis

Sample size estimation was performed during the ethics approval process. Assuming a two-sided alpha level of 0.05, 80% statistical power, and an expected effect size of 0.30, corresponding to an approximately 20–30% difference in 5-year survival between clinically relevant risk groups, based on previously reported data in stage III NSCLC [3], the minimum required sample size was estimated to be approximately 200–240 patients. The final cohort included 390 eligible patients, exceeding this minimum requirement, and was therefore considered adequate for the planned survival and prognostic analyses.

Continuous variables were summarized as mean ± standard deviation or median with interquartile range, as appropriate, and categorical variables as frequencies and percentages. Normality was assessed using the Kolmogorov–Smirnov test. Survival analyses were performed using the Kaplan–Meier method, with comparisons by the log-rank test.

OS was defined as the time from surgery to death from any cause or to last follow-up, and RFS was defined as the time from surgery to the first documented recurrence. Patients without events were censored at the last follow-up.

Univariate Cox proportional hazards regression was used to identify factors associated with OS and RFS. Variables with *p* < 0.10 were entered into multivariable Cox models, which were constructed using backward elimination guided by clinical relevance and statistical significance. Hazard ratios with 95% confidence intervals were reported.

For RFS, pathologic N classification was not included in the final multivariable model because it did not retain independent prognostic significance when analyzed together with disease stage and histopathological diagnosis; therefore, diagnosis- and stage-based variables were retained.

Proportional hazards assumptions were assessed using Schoenfeld residuals, and model fit was evaluated with the omnibus test. Model discrimination was quantified using Harrell’s concordance index (C-index) with 95% confidence intervals derived from bootstrap resampling (200 iterations). Calibration was assessed using plots comparing predicted and observed survival at predefined time points.

Nomograms were developed based on independent prognostic factors to estimate 12-, 36-, and 60-month OS and 12-, 24-, and 36-month RFS probabilities. All analyses were two-sided, with *p* < 0.05 considered statistically significant, and were performed using SPSS version 27.0 and Python version 3.12.

### 2.6. Use of Artificial Intelligence

Generative artificial intelligence tools were used solely for the preparation and visual enhancement of graphical elements. These tools were not used for data analysis, statistical modeling, or interpretation of results.

## 3. Results

### 3.1. Patient Characteristics

A total of 390 stage III patients who underwent upfront surgery for NSCLC between January 2009 and December 2023 were included in the study. The majority of patients were male, and the mean age was in the mid-60s. The most common histological subtype was squamous cell carcinoma. The most frequently performed surgical procedure was pneumonectomy.

According to the 9th TNM classification, the majority of patients were stage IIIA, while there were no stage IIIC patients. All cases underwent systematic mediastinal lymph node dissection, and the pathological node status ranged from N0 to N2b. Most patients received adjuvant chemotherapy, and selected cases received adjuvant radiotherapy. Postoperative complications were observed in a significant proportion of patients. Detailed information on the baseline characteristics of the patients is presented in Table 1.

Despite preoperative exclusion of multi-station N2 disease, a subset of patients was found to have pathological multi-station N2 involvement, indicating a clinically relevant rate of nodal upstaging.

### 3.2. Survival Outcomes

**Figure 2:**
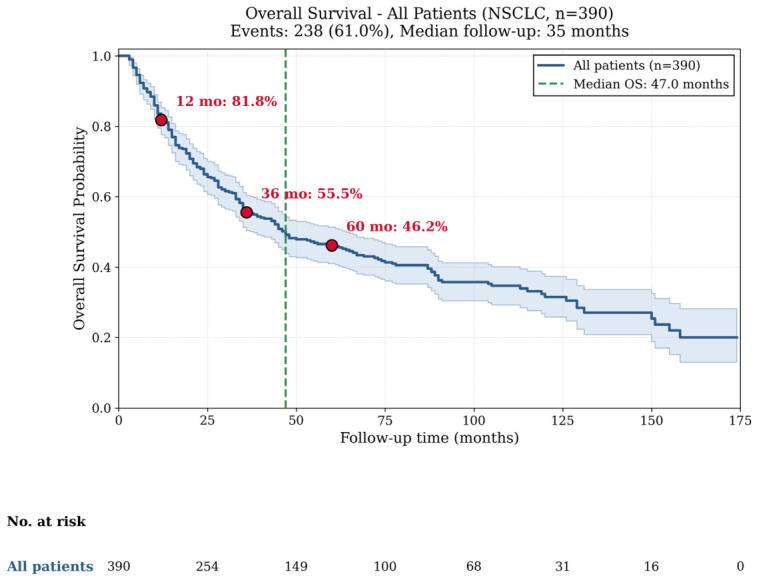
Kaplan–Meier curve showing OS in patients with stage III NSCLC treated with upfront surgical resection.

**Figure 3:**
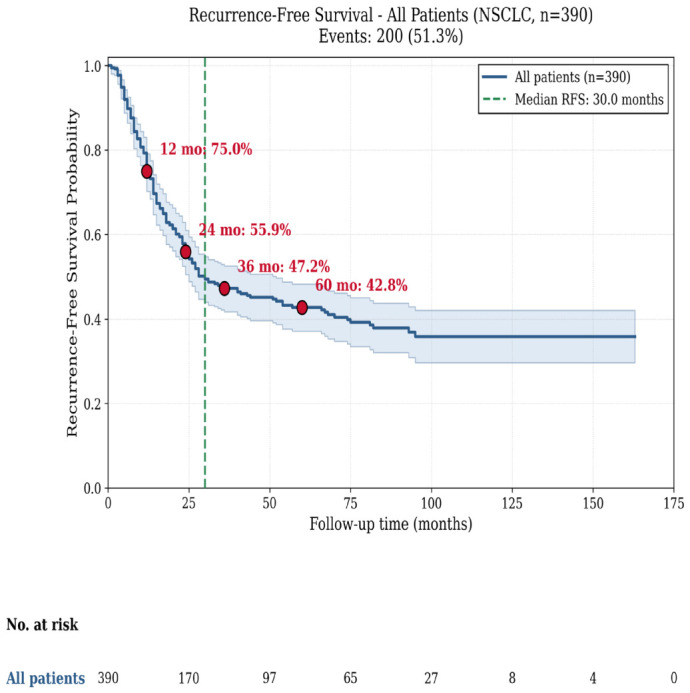
Kaplan–Meier curve showing RFS after upfront surgery in patients with stage III NSCLC.

Survival analyses were performed after an average follow-up period of 35 months. The median OS was 47 months (95% CI, 33.7–60.3 months). The median RFS was 30 months (95% CI, 17–43 months). Kaplan–Meier curves for OS and RFS are shown in Figure 2 and Figure 3.

### 3.3. Prognostic Factors for OS

Univariable Cox regression analysis identified age, PET-detected N2, tumor location, postoperative complications, pathologic nodal status, adjuvant chemotherapy, and adjuvant radiotherapy as factors significantly associated with OS.

In multivariable analysis, increasing age remained independently associated with worse OS. PET-detected N2, left-sided tumor location, and postoperative complications were also independently associated with poorer survival. Higher pathologic nodal categories (pN1 and pN2b) were associated with worse OS compared with pN0, whereas pN2a did not retain independent significance. Adjuvant chemotherapy was associated with improved OS, while adjuvant radiotherapy was associated with worse OS (Table 2).

### 3.4. Prognostic Factors for RFS

In univariable analysis, PET-detected N2, tumor location, histopathological subtype, pathologic nodal status, disease stage, and adjuvant radiotherapy were significantly associated with RFS.

In multivariable analysis, tumor location remained an independent prognostic factor, with left-sided tumors associated with worse RFS. Histopathological subtype also remained significant, with adenocarcinoma associated with a higher risk of recurrence than squamous cell carcinoma. Pathologic stage IIIB disease was independently associated with worse RFS compared with stage IIIA. Although adjuvant radiotherapy showed a trend toward worse RFS, this association did not reach statistical significance. Pathologic nodal subgroups and PET-detected N2 did not retain independent prognostic significance in the final model (Table 3).

### 3.5. Nomogram Development and Performance

Nomograms were constructed based on independent prognostic factors identified in the multivariable Cox models to estimate individualized survival probabilities. The final multivariable Cox model was statistically significant (omnibus test, χ^2^ = 80.77; df = 12; *p* < 0.001).

**Figure 4:**
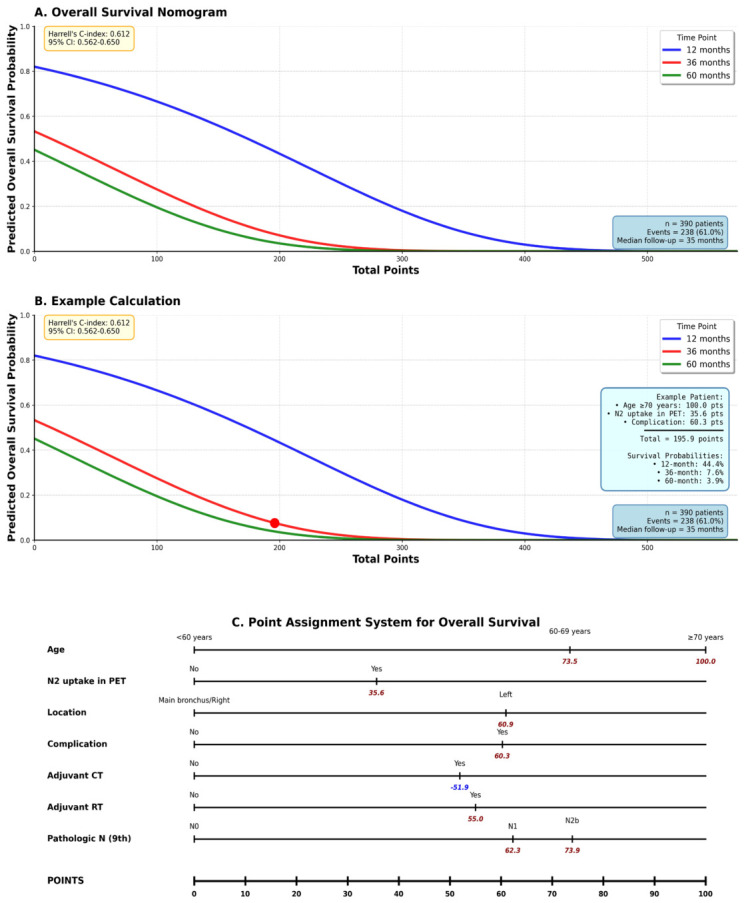
OS nomogram for stage III NSCLC. (**A**) Nomogram predicting 12-, 36-, and 60-month OS based on independent prognostic factors from the multivariable Cox model. (**B**) Example calculation demonstrating individualized survival estimation using nomogram points. (**C**) Point assignment system for each prognostic variable. Model discrimination is summarized by the C-index (0.612; 95% CI, 0.562–0.650). Additional calibration plots and exploratory risk stratification analyses are provided in Supplementary Figures S1 and S2, respectively. PET, positron emission tomography; CT, chemotherapy; RT, radiotherapy.

The OS nomogram predicted 12-, 36-, and 60-month survival probabilities and demonstrated moderate discriminative ability, with a Harrell’s C-index of 0.612 (95% CI: 0.562–0.650). Calibration analysis at 36 months showed systematic underestimation of observed survival probabilities (Figure 4).

**Figure 5:**
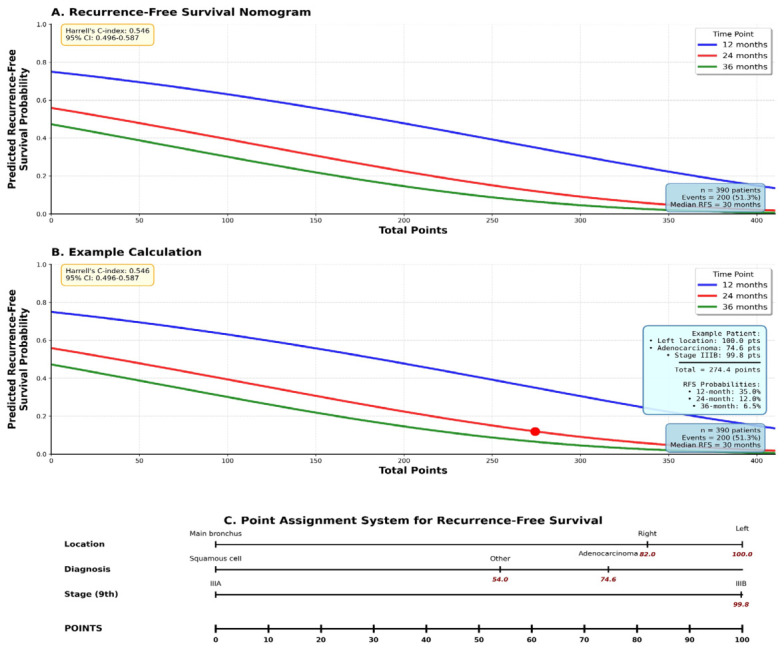
RFS nomogram for stage III NSCLC. (**A**) Nomogram predicting 12-, 24-, and 36-month RFS based on independent prognostic factors. (**B**) Example calculation demonstrating individualized RFS estimation. (**C**) Point assignment system for each prognostic variable. Model discrimination is summarized by the C-index (0.546; 95% CI, 0.496–0.587). Additional calibration plots and exploratory risk stratification analyses are provided in Supplementary Figures S1 and S2, respectively.

The RFS nomogram predicted 12-, 24-, and 36-month RFS probabilities but demonstrated limited discriminative performance (C-index 0.546; 95% CI: 0.496–0.587). Calibration analysis at 24 months showed variable and suboptimal agreement between predicted and observed RFS (Figure 5).

## 4. Discussion

This study evaluated long-term survival outcomes and prognostic factors in patients with stage III NSCLC who underwent surgical treatment. Our findings suggest that, in carefully selected patients, surgical intervention may contribute to long-term survival, although these results should be interpreted in the context of patient selection, disease characteristics, and treatment strategies. This interpretation is particularly important given the exclusion of early postoperative mortality from the analysis. In addition, overall survival outcomes should be interpreted as conditional survival beyond the early postoperative period, as patients who died within 90 days after surgery were excluded from the analysis. The better survival outcomes with R0 resection and limited lymph node involvement suggest that surgery is not merely a means of local control but can be an effective component of a multimodal treatment. These findings highlight the limitations of uniform treatment algorithms in stage III NSCLC. The relatively high pneumonectomy rate in this cohort reflects the inclusion of carefully selected patients with centrally located or locally advanced tumors requiring extended resections, particularly in cases where complete resection could not be achieved with lesser procedures, and should be interpreted as a reflection of case selection rather than a deviation from standard surgical practice.

This study reflects a historical cohort derived from the pre-immunotherapy era, which should be taken into account when interpreting the results. In recent years, the therapeutic landscape of resectable NSCLC has evolved significantly with the introduction of immunotherapy. Neoadjuvant immunochemotherapy has been shown to improve pathological response and survival outcomes compared to chemotherapy alone, while perioperative strategies further enhance long-term outcomes without increasing surgical risk [12,13]. Real-world data suggest that these benefits are particularly pronounced in stage III and N2 disease [14]. However, despite these advances, the optimal treatment strategy remains unclear. Recent analyses indicate that perioperative immunotherapy does not provide a clear survival advantage over neoadjuvant approaches, highlighting the importance of appropriate patient selection [15]. In this context, our study provides baseline data from the pre-immunotherapy era, allowing a clearer understanding of the prognostic impact of surgical and nodal factors and facilitating comparison with contemporary multimodal strategies. Taken together, these findings emphasize that while systemic therapies continue to evolve, the biological heterogeneity of stage III NSCLC necessitates individualized, multidisciplinary treatment strategies, in which both tumor biology and surgical factors remain critical determinants.

The survival results from our study suggest a re-evaluation of surgery in the era of immunotherapy and reveal that this observation is not entirely new in the literature. Indeed, numerous retrospective studies published before the immunotherapy era have reported that surgical intervention can provide significant long-term survival in carefully selected patients with stage III NSCLC [6,16,17]. These studies highlighted limited mediastinal lymph node involvement, R0 resection, and the use of adjuvant therapy as key determinants of survival. Previous studies have shown that surgical intervention can provide meaningful long-term survival in carefully selected stage III NSCLC patients, emphasizing the importance of patient selection and tumor biology [7,8]. The heterogeneity observed in this cohort reflects real-world clinical practice rather than a limitation of the study design.

Prior to the advent of immunotherapy, upfront surgical intervention was considered an appropriate strategy for carefully selected patients, especially those exhibiting limited mediastinal nodal involvement and favorable clinical profiles. In the age of immunotherapy, however, the positive impact of consolidation immunotherapy following concomitant chemoradiotherapy on survival has significantly shaped treatment standards for stage III NSCLC. Significant gains in progression-free survival in the primary analysis of the PACIFIC study, and in OS in its updated analyses, led to the adoption of durvalumab consolidation after concomitant chemoradiotherapy as the standard treatment for this patient group [18,19]. Based on this evidence, current international guidelines have highlighted non-surgical multimodal approaches, particularly in unresectable stage III disease, and recommend durvalumab consolidation following concomitant chemoradiotherapy as the standard treatment [1,2,3]. However, it should be noted that patients considered suitable for surgical resection were not represented in the PACIFIC trial, and therefore its findings mainly reflect outcomes in patients with unresectable disease. The differences between the selected structure of clinical study populations and real-life applications reveal that, even in the age of immunotherapy, there is significant heterogeneity in patient profiles, access to treatment, toxicity, and treatment continuity [20,21]. In this context, the role of surgery in the age of immunotherapy should be considered not as an alternative to the standard approaches defined in guidelines, but as an approach that can be applied in carefully selected patient groups after multidisciplinary evaluation and that complements multimodal therapy.

Recent real-world data on unresectable stage III NSCLC treated with concurrent chemoradiotherapy and consolidation immunotherapy have demonstrated that survival outcomes remain heterogeneous and are significantly influenced by histological subtype, with squamous cell carcinoma associated with poorer survival compared to adenocarcinoma [22]. In this context, the relatively favorable outcomes observed in selected surgical cohorts further emphasize the importance of appropriate patient selection and multidisciplinary decision-making.

The observed association between adjuvant radiotherapy and worse overall survival in our cohort should be interpreted with caution. In our study, radiotherapy was administered primarily to patients with pathologically confirmed N2 disease, representing a subgroup with an inherently worse prognosis. The role of postoperative radiotherapy in N2 NSCLC remains controversial. A recent meta-analysis demonstrated improved overall and disease-free survival with the addition of radiotherapy following adjuvant chemotherapy in patients with resected N2 disease [23]. In contrast, the phase III LungART trial showed that postoperative radiotherapy did not significantly improve disease-free or overall survival, although it reduced locoregional recurrence [24]. Taken together, these findings suggest that the impact of radiotherapy is highly dependent on patient selection, and the apparent negative association observed in retrospective cohorts likely reflects differences in disease severity rather than a true detrimental effect of radiotherapy.

The role of radiotherapy in stage III NSCLC is closely linked to accurate staging and treatment planning. In this context, 18F-FDG PET-CT has emerged as a critical tool not only for disease staging but also for optimizing radiotherapy target delineation. Previous studies have demonstrated that PET-CT significantly improves target volume delineation and alters treatment planning by enhancing the detection of tumor margins and nodal involvement, leading to more precise radiotherapy delivery [25]. Furthermore, PET/CT-based image-guided radiotherapy approaches have been shown to be feasible and safe even in high-risk patient populations with compromised pulmonary function [26]. More recently, PET-CT-guided volume reduction strategies have been associated with decreased treatment-related toxicity, improved treatment compliance, and increased likelihood of successful transition to consolidation immunotherapy, highlighting its growing importance in modern multimodal treatment strategies for stage III NSCLC [27].

One of the main reasons why surgical approaches are controversial in stage III NSCLC is that the concepts of “resectable” and “unresectable” are often used interchangeably in clinical practice, as is the distinction between “operable” and “inoperable.” However, resectability describes the complete anatomical and oncological removal of the disease, while operability refers to the physiological and clinical factors specific to the patient. Current consensus documents and systematic reviews clearly show that there is no clear boundary between these two concepts in stage III disease, and that the decision on resectability can vary depending on the center’s experience, the surgeon’s approach, and the multidisciplinary team’s assessment [4,28]. Indeed, real-life data showing that patients with the same clinical stage are managed with surgery, neoadjuvant therapy, or non-surgical multimodal approaches in different centers suggest that standard algorithms alone are insufficient for treatment selection [29]. The discrepancy between clinical and pathological nodal staging highlights the inherent limitations of current staging techniques, even with modern imaging and invasive staging methods. In this context, surgical decisions in stage III NSCLC should not be based solely on guideline-based classifications, but rather on a multidisciplinary decision-making process that considers the anatomical spread characteristics of the disease, the nodal involvement pattern, and patient-specific clinical factors [5]. The fact that the group of patients who underwent surgery in our study was determined as a result of such a selective and multidimensional evaluation provides a critical context in interpreting the obtained survival results.

In recent years, numerous studies have used nomograms and risk models to predict survival in patients with stage III NSCLC. Although these studies have used large patient series and advanced statistical methods, they have shown limited discriminatory power for clinical decision-making. In particular, the generally moderate C-index values and inconsistent calibration performance limit the use of these tools as definitive predictors at the individual patient level [9,10,11]. The nomogram developed in our study also demonstrated moderate discrimination for OS (C-index = 0.612), reflecting the significant clinical and anatomical heterogeneity of stage III NSCLC. Inclusion of variables reflecting real-world data, such as postoperative complications and nodal involvement patterns, may have increased clinical applicability but inevitably limited predictive discrimination. This observation suggests that survival in stage III NSCLC has a complex biological background that cannot be fully explained by single clinical–pathological parameters, and that model performance is naturally limited in this context. Therefore, nomograms should be considered as guiding and complementary tools in clinical risk stratification. Conversely, they should not replace multidisciplinary clinical assessment and individualized patient selection in critical decisions such as determining surgical candidacy. The findings of our study also support the importance of holistic decision-making processes that consider the anatomical and biological characteristics of the disease, rather than relying solely on model-based approaches. The aim of this study is not to challenge guideline-recommended treatment strategies, but to provide a real-world perspective on the heterogeneity of stage III NSCLC and to emphasize the importance of individualized treatment approaches. The fact that the nomogram developed for RFS shows a more limited discriminatory performance and its calibration is variable suggests that RFS in stage III NSCLC is determined not only by tumor burden and anatomical factors, but also by dynamic processes that are difficult to measure clinically, such as micrometastatic disease, biological aggressiveness, and response to adjuvant therapy. Therefore, the weaker modeling performance for RFS compared to OS should be considered an expected finding reflecting the biological complexity of the disease, rather than a methodological deficiency. This finding necessitates cautious use of formal risk classifications for RFS in clinical decision-making.

The developed nomogram may provide a framework for individualized risk estimation; however, given its moderate discrimination, it should be considered exploratory and requires external validation.

This study has several limitations, including its retrospective design, single-center setting, and inherent selection bias, which may limit generalizability. Moreover, the focus on surgically treated patients does not capture the full spectrum of stage III NSCLC. In addition, although operative and pathology reports were reviewed, lymph node reporting was not fully standardized across the study period. In some cases, only positive lymph nodes were documented, while negative lymph nodes were not consistently reported. This variability limited the reliability of quantitative lymph node parameters and precluded their inclusion in statistical analyses. Nevertheless, these real-world data indicate that, even in the immunotherapy era, treatment decisions in stage III disease remain highly dependent on individualized, multidisciplinary evaluation. Our findings support surgery as a complementary component of multimodal treatment in carefully selected patients, rather than an alternative to guideline-recommended approaches.

## 5. Conclusions

Stage III NSCLC demonstrates substantial heterogeneity in survival outcomes even within surgically treated cohorts. While upfront surgery remains a viable option in selected patients, treatment decisions should be guided by individualized risk assessment and multidisciplinary evaluation rather than uniform staging-based algorithms. Prognostic models may provide supportive information but should complement, not replace, clinical judgment.

## Figures and Tables

**Table 1:** Baseline Clinicopathological Characteristics.

Characteristics	Overall (*n* = 390)
Age	62.5 ± 8.7
Mass SUV_max_	15.6 ± 6.7
Mass diameter (cm)	6.8 ± 3.1
Post-operative hospital stay (days)	7.8 ± 6.2
Gender	
Male	358 (91.8)
Female	32 (8.2)
Smoking status	
Never	29 (7.4)
Former	119 (30.5)
Current	242 (62.1)
ECOG performance status	
0–1	369 (94.6)
≥2	21 (5.4)
N1 in PET	
No	260 (66.7)
Yes	130 (33.3)
N2 in PET	
No	224 (57.4)
Yes	166 (42.6)
Endobronchial lesion	
No	196 (50.3)
Yes	194 (49.7)
Location	
Main bronchus	78 (20)
Right	158 (40.5)
Left	154 (39.5)
Operation	
Pneumonectomy	204 (52.3)
Lobectomy	186 (47.7)
Complication ^a^	
No	308 (79)
Yes	82 (21)
Reoperation ^b^	
No	356 (91.3)
Yes	34 (8.7)
Histopathology	
Squamous cell carcinoma	236 (60.5)
Adenocarcinoma	120 (30.8)
Other	34 (8.7)
Invasion of anatomical structures	
No	202 (51.8)
Yes	188 (48.2)
Pathological N1 positivity	
No	146 (37.4)
Yes	244 (62.6)
pT (9th)	
T1–2	48 (12.3)
T3	114 (29.2)
T4	228 (58.5)
pN (9th)	
N0	86 (22.1)
N1	178 (45.6)
N2a	78 (20)
N2b	48 (12.3)
Stage (9th)	
IIIA	333 (85.4)
IIIB	57 (14.6)
Adjuvant chemotherapy	
No	66 (16.9)
Yes	324 (83.1)
Adjuvant radiotherapy	
No	298 (76.4)
Yes	92 (23.6)
Recurrence	
No	190 (48.7)
Yes	200 (51.3)
Survival	
Alive	152 (39)
Dead	238 (61)

^a^ Any postoperative complications, minor or major; ^b^ Reoperation due to a major complication such as bleeding or bronchopleural fistula. Data are presented as n (%) for categorical variables and as mean ± standard deviation for continuous variables. PET, positron emission tomography; SUVmax, maximum standardized uptake value.

**Table 2:** Univariable and Multivariable Cox Proportional Hazards Analysis for OS.

	Univariable			Multivariable		
Variables	β Coefficient	*p*	HR (95% CI)	β Coefficient	*p*	HR (95% CI)
Age						
<60 years	Reference	-	1	Reference	-	1
60–69 years	0.71	0.001	2.03 (1.48–2.80)	0.65	0.001	1.91 (1.37–2.65)
≥70 years	1.09	0.001	2.96 (2.06–4.26)	0.88	0.001	2.41 (1.66–3.49)
Mass SUV_max_	−0.01	0.209	0.99 (0.97–1.01)			
Mass diameter (cm)	−0.03	0.207	0.97 (0.93–1.02)			
Gender						
Male	Reference	-	1			
Female	0.28	0.208	1.33 (0.85–2.06)			
N1 in PET						
No	Reference	-	1	Reference	-	1
Yes	0.36	0.007	1.44 (1.10–1.87)	0.19	0.227	1.20 (0.89–1.63)
N2 in PET						
No	Reference	-	1	Reference	-	1
Yes	0.42	0.001	1.53 (1.18–1.98)	0.31	0.029	1.37 (1.03–1.81)
Endobronchial lesion						
No	Reference	-	1			
Yes	−0.14	0.283	0.87 (0.67–1.12)			
Location						
Main bronchus	Reference	-	1	Reference	-	1
Right	0.31	0.107	1.36 (0.94–1.96)	0.24	0.228	1.28 (0.86–1.90)
Left	0.47	0.012	1.599 (1.11–2.31)	0.54	0.006	1.71 (1.17–2.50)
Operation						
Pneumonectomy	Reference	-	1			
Lobectomy	0.06	0.631	1.07 (0.83–1.37)			
Complication						
No	Reference	-	1	Reference	-	1
Yes	0.33	0.032	1.39 (1.03–1.88)	0.53	0.002	1.70 (1.21–2.37)
Reoperation						
No	Reference	-	1			
Yes	0.24	0.284	1.27 (0.82–1.98)			
Histopathology						
Other	Reference	-	1			
Squamous cell carcinoma	0.20	0.458	1.22 (0.73–2.04)			
Adenocarcinoma	0.22	0.423	1.25 (0.73–2.14)			
Invasion of anatomical structures						
No	Reference	-	1			
Yes	−0.11	0.401	0.90 (0.70–1.160)			
Pathological N1 positivity						
No	Reference	-	1			
Yes	0.24	0.078	1.27 (0.97–1.66)			
pT (9th)						
T1–2	Reference	-	1			
T3	−0.02	0.944	0.99 (0.64–1.51)			
T4	−0.12	0.554	0.89 (0.60–1.32)			
pN (9th)						
N0	Reference	-	1	Reference	-	1
N1	0.56	0.002	1.74 (1.22–2.49)	0.55	0.003	1.73 (1.20–2.49)
N2a	0.44	0.036	1.56 (1.03–2.36)	0.17	0.460	1.18 (0.76–1.83)
N2b	0.78	0.001	2.19 (1.37–3.48)	0.65	0.009	1.91 (1.17–3.12)
Stage (9th)						
IIIA	Reference	-	1			
IIIB	0.28	0.115	1.33 (0.93–1.88)			
Adjuvant chemotherapy						
No	Reference	-	1	Reference	-	1
Yes	−0.42	0.010	0.66 (0.48–0.91)	−0.46	0.010	0.63 (0.45–0.90)
Adjuvant radiotherapy						
No	Reference	-	1	Reference	-	1
Yes	0.41	0.004	1.50 (1.14–1.99)	0.48	0.004	1.62 (1.17–2.25)

Variables with *p* < 0.10 in univariate analysis were entered into the multivariable Cox regression model. *p* < 0.05 was considered statistically significant. CI = confidence interval; HR = hazard ratio; PET = positron emission tomography; SUVmax = maximum standardized uptake value.

**Table 3:** Univariable and Multivariable Cox Proportional Hazards Analysis for RFS.

	Univariable			Multivariable		
Variables	β Coefficient	*p*	HR (95% CI)	β Coefficient	*p*	HR (95% CI)
Age						
<60 years	Reference	0.478	1			
60–69 years	0.18	0.263	1.19 (0.88–1.63)			
≥70 years	0.02	0.942	1.016 (0.67–1.54)			
Mass SUV_max_	−0.01	0.499	0.99 (0.97–1.01)			
Mass diameter (cm)	0.01	0.529	1.01 (0.97–1.06)			
Gender						
Male	Reference	-	1			
Female	0.17	0.489	1.19 (0.73–1.93)			
N1 in PET						
No	Reference	-	1			
Yes	0.25	0.091	1.28 (0.96–1.72)			
N2 in PET						
No	Reference	-	1	Reference	-	1
Yes	0.31	0.033	1.36 (1.03–1.79)	0.18	0.248	1.19 (0.88–1.61)
Endobronchial lesion						
No	Reference	-	1			
Yes	0.24	0.096	1.27 (0.96–1.68)			
Location						
Main bronchus	Reference	-	1	Reference	-	1
Right	0.56	0.008	1.75 (1.16–2.65)	0.39	0.084	1.47 (0.95–2.29)
Left	0.51	0.017	1.67 (1.09–2.53)	0.47	0.029	1.60 (1.05–2.45)
Operation						
Pneumonectomy	Reference	-	1			
Lobectomy	−0.26	0.072	0.78 (0.59–1.02)			
Complication						
No	Reference	-	1			
Yes	0.03	0.880	1.03 (0.72–1.46)			
Reoperation						
No	Reference	-	1			
Yes	−0.05	0.858	0.95 (0.57–1.59)			
Histopathology						
Squamous cell carcinoma	Reference	-	1	Reference	-	1
Adenocarcinoma	0.37	0.015	1.45 (1.07–1.94)	0.35	0.027	1.42 (1.04–1.95)
Other	0.28	0.267	1.327 (0.81–2.19)	0.26	0.334	1.29 (0.77–2.16)
Invasion of anatomical structures						
No	Reference	-	1			
Yes	−0.13	0.365	0.88 (0.67–1.16)			
Pathological N1 positivity						
No	Reference	-	1			
Yes	0.12	0.408	1.13 (0.85–1.50)			
pT (9th)						
T1–2	Reference	-	1			
T3	−0.31	0.204	0.73 (0.45–1.19)			
T4	0.07	0.745	1.07 (0.70–1.64)			
pN (9th)						
N0	Reference	-	1			
N1	0.21	0.265	1.24 (0.85–1.80)			
N2a	0.17	0.453	1.18 (0.76–1.83)			
N2b	0.79	0.001	2.20 (1.37–3.52)			
Stage (9th)						
IIIA	Reference	-	1	Reference	-	1
IIIB	0.60	0.001	1.82 (1.28–2.58)	0.47	0.016	1.60 (1.09–2.35)
Adjuvant chemotherapy						
No	Reference	-	1			
Yes	−0.01	0.980	0.99 (0.68–1.45)			
Adjuvant radiotherapy						
No	Reference	-	1	Reference	-	1
Yes	0.41	0.010	1.50 (1.10–2.05)	0.32	0.060	1.38 (0.99–1.93)

Variables with *p* < 0.10 in univariate analysis were entered into the multivariable Cox regression model. *p* < 0.05 was considered statistically significant. CI = confidence interval; HR = hazard ratio; PET = positron emission tomography; SUVmax = maximum standardized uptake value.

## Data Availability

The data supporting the findings of this study are available from the corresponding author upon reasonable request.

## Supplementary Materials

The following supporting information can be downloaded at: https://www.mdpi.com/article/10.3390/curroncol33060316/s1, Figure S1: Calibration plots of the nomogram models for OS (A) and RFS (B), showing agreement between predicted and observed survival probabilities; Figure S2: Exploratory risk stratification based on total nomogram points. Kaplan–Meier curves show survival by nomogram-derived risk groups. This analysis is exploratory.

## Abbreviations

The following abbreviations are used in this manuscript:

NSCLC	Non-Small Cell Lung Cancer
R0	Complete Resection
TNM	Tumor–Node–Metastasis
PET	Positron Emission Tomography
OS	Overall Survival
RFS	Recurrence-free Survival
