# Supplementary figures and images for: Prognostic Factors and Survival in Surgically Treated Stage III Non-Small Cell Lung Cancer: A Real-World Single-Center Retrospective Cohort Study
Source: Curr Oncol. 2026 May 28;33(6):316. doi: 10.3390/curroncol33060316 (PMC13297716; doi:10.3390/curroncol33060316)

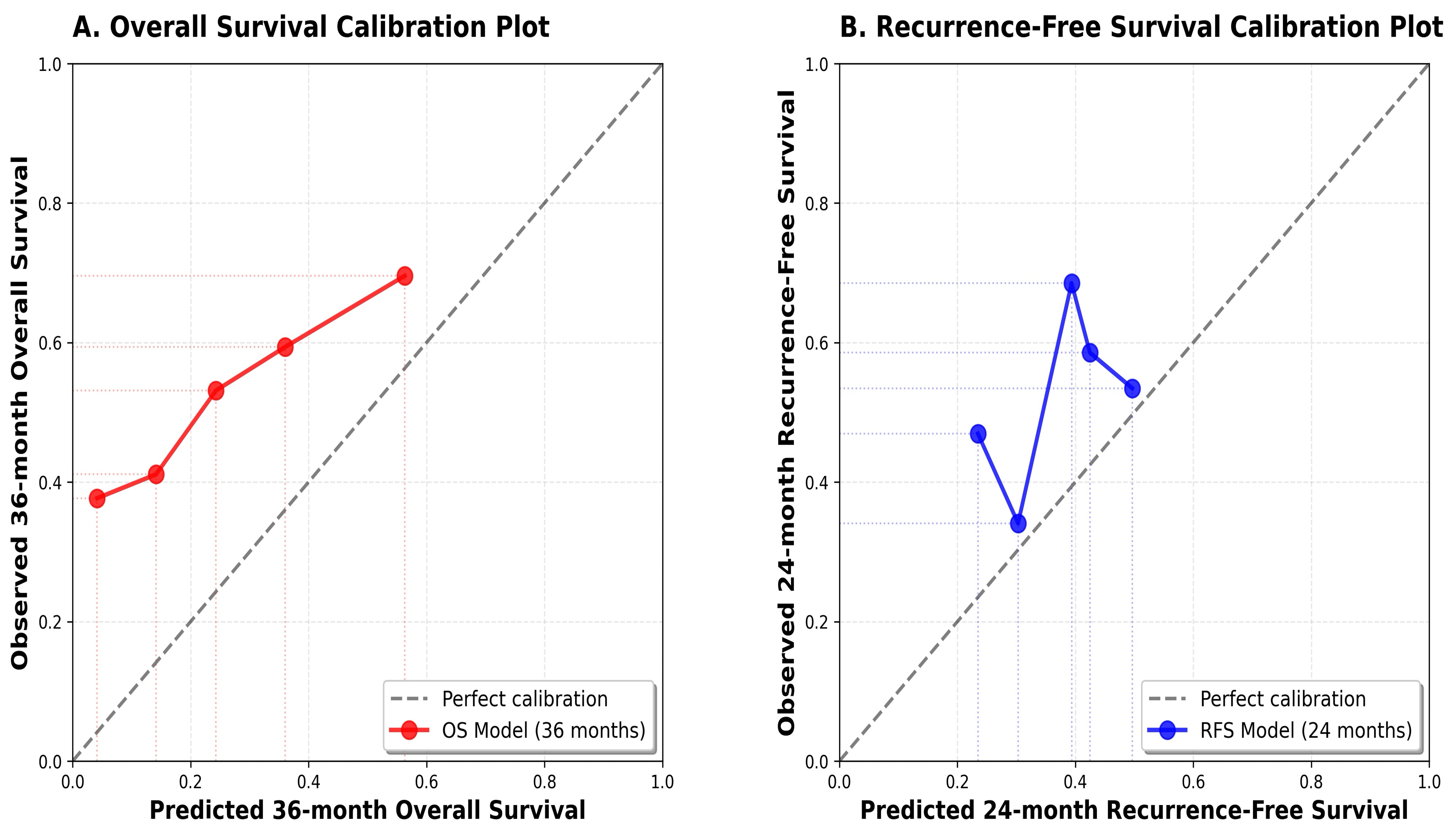

Supplement: Supplementary file 1 [file curroncol-33-00316-s001.zip › Supplementary Figure S1. Calibration plots.tiff]

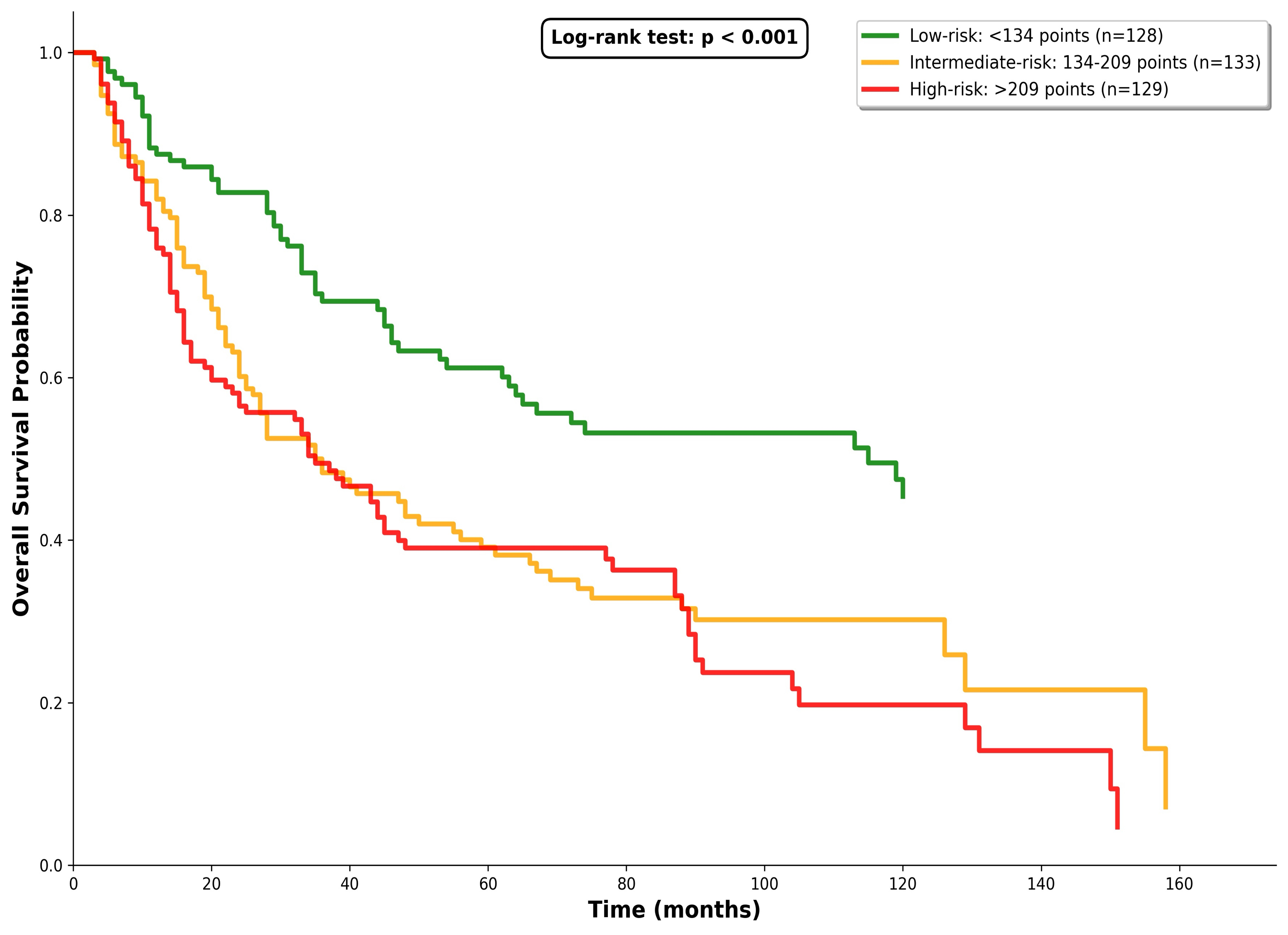

Supplement: Supplementary file 1 [file curroncol-33-00316-s001.zip › Supplementary Figure S2. Risk stratification.tiff]
